# Development of a Human Electrophysiology Laboratory to Advance Cognitive Neuroscience in Western Kenya

**DOI:** 10.1177/15500594261432722

**Published:** 2026-05-11

**Authors:** Amira Nafiseh, Jaime Morales, Mark Nyalumbe, Eren Oyungu, David Ayuku, Amanda R Bolbecker, William P Hetrick, Megan S McHenry

**Affiliations:** 1 Richard M. Fairbanks School of Public Health, 8785 Indiana University, Indianapolis, USA; 2 Department of Psychological and Brain Sciences, 1772 Indiana University, Bloomington, USA; 3 Program in Neuroscience, 8785 Indiana University, Bloomington, USA; 4 107853 Academic Model Providing Access to Healthcare, Eldoret, Kenya; 5 Department of Child Health, Moi University School of Medicine; 6 Department of Mental Health and Behavioral Sciences, Moi University School of Medicine, Eldoret, Kenya; 7 Department of Psychiatry, 12250 Indiana University School of Medicine, Indianapolis, USA; 8 Department of Pediatrics, 12250 Indiana University School of Medicine, Indianapolis, USA

**Keywords:** neurophysiology, electroencephalography, cognitive neuroscience, public health infrastructure, LMIC, global health

## Abstract

Increasing evidence underscores the influence of social determinants and cultural factors on brain structure and function. Neurophysiological approaches offer unique advantages for assessing brain physiology and cognitive functioning, but challenges remain in building the capacity necessary to support the use of these resources in low- and middle-income countries (LMICs). The establishment of a first-of-its-kind neurophysiology lab in East Africa in January 2022, the Moi University Clinical and Cognitive Neuroscience Center (MU CCNC), promotes dedicated research infrastructure for patients within Moi Teaching and Referral Hospital (MTRH), the second-largest national referral hospital in Kenya, which serves about 24 million individuals. Built within the Academic Model Providing Access to Healthcare (AMPATH), a three-decade long collaboration between MTRH and a consortium of North American universities, the critical lessons learned while building this infrastructure in a low-resource setting included overcoming barriers to using and maintaining highly sensitive equipment in a setting with substantial environmental noise and electrical interference, ensuring secure and accurate data storage for complex files from precisely synchronized programs, and establishing standard operating procedures for laboratory continuity. Within the first two years of the establishment of the lab, capacity building has been prioritized, with a focus on extending neurophysiology training to research staff, students, and faculty throughout Kenya and developing research questions that can be addressed with lab methods and resources. By describing the process of developing a comprehensive human electrophysiology lab in a LMIC, we hope to streamline implementation for researchers to establish neurophysiology infrastructure in similar settings.

## Introduction

Despite remarkable strides made in the field of human neuroscience, significant knowledge gaps remain with respect to understanding the neural processes associated with neurocognitive function and mental health. One substantial gap lies in comprehending how these neural processes may vary among different global populations, particularly those residing in low- and middle-income countries (LMICs). Astonishingly, despite 85% of the world's population residing in LMICs, there is a glaring scarcity of neuroscience research originating from these regions.^1,2^ Considering the profound impact that individual, environmental, and social determinants of health and cultural factors wield over development, epigenetics of neurological processes, and mental health, these factors represent a substantial impediment to our progress in comprehending brain function across diverse global populations.^3-5^

One of the predominant factors contributing to this gap in neuroscience is the lack of infrastructure for conducting human neuroscience research in LMICs.^6^ Some cutting-edge neuroimaging approaches, like functional magnetic resonance imaging (fMRI), require expensive equipment and highly trained technicians, making them primarily accessible in high-income countries (HICs).^7-9^ However, other neuroimaging methods, such as electroencephalography (EEG), offer less financially costly and non-invasive means of assessing neural function. EEG, in particular, is a valuable tool to assess cognition, beginning with early sensory processing and extending to later stages of cognitive operations when event-related brain potentials (ERPs) are measured,^10^ with the potential to assess psychophysiological processes and identify potential biomarkers and therapeutic targets.

Fortunately, international organizations and governmental funding agencies are actively dedicating resources to the development of research infrastructure.^11,12^ Nevertheless, establishing new research facilities for human neuroscience, particularly those involving neuroimaging techniques like EEG, presents a formidable challenge. While there are existing descriptions of how to set up an EEG laboratory, none of them are tailored to the unique considerations required in LMICs.^13^ Establishing a comprehensive neurophysiology lab in such settings demands a range of specific considerations, including ensuring basic infrastructure like reliable electricity, minimizing ambient noise in open-air buildings, provision of appropriate technical training, and optimizing laboratory design.

To address this gap, we describe the process undertaken to establish a neurophysiology laboratory within Moi University in Eldoret, Kenya, with a specific focus on strategies used to overcome challenges common in LMICs. We discuss various aspects of our process, including laboratory design, equipment procurement, and, briefly, the training of local research personnel to troubleshoot technical issues and collect high-quality EEG and ERP data in a resource-constrained environment. By sharing our experiences developing this laboratory, we hope to facilitate and streamline the process for other clinical and cognitive neuroscience researchers.

## Methods

### Setting

This first-of-its-kind neurophysiology laboratory is housed in Moi University's Medical Education Center as part of the Academic Model Providing Access to Healthcare (AMPATH), an ongoing collaboration established over 30 years ago between Moi University and Moi Teaching and Referral Hospital (MTRH) in Eldoret, Kenya, where the partnership is based, and a consortium of North American universities led by Indiana University (IU). AMPATH was established in 1990 as a bilateral exchange program, initially known as the IU-Kenya Partnership, with a tripartite mission focused on advancing clinical care, education, and research in partnership with Kenyan counterparts.^14^

In 2010, the AMPATH Mental Working Group—consisting of Kenyan, North American, and European mental health experts—was formed, contributing to increased interest in the biological aspects of mental health and neuroscience methods across of faculty, students, and graduates. By 2019, the groundwork for the establishment of the Moi University Clinical and Cognitive Neuroscience Center (MU CCNC) was laid, based on bilateral interest in facilitating the promotion of clinical and cognitive neuroscience methods to serve research and training needs. The opening of the MU CCNC in early 2022 enhanced research capacity within the partnership and created a dedicated space for projects relevant to patients cared at MTRH—the second largest national referral hospital in Kenya serving about 24 million individuals in western Kenya, eastern Uganda, and southern Sudan.^15^

### Team Composition

AMPATH-based North American and Kenyan faculty spearheaded the development of this neurophysiology lab, with key collaboration between pediatricians with expertise in neurology and physiology and chairpersons of the respective universities’ psychology departments. Moi faculty led efforts to establish a secure and appropriate space for the lab, as well as support from the university. North American faculty led efforts to optimize the lab space, minimize electrical and environmental interference, and oversee equipment set-up and maintenance. Key personnel, including clinical psychology and neuroscience PhD students, an EEG specialist, a research scientist, and research technicians, helped with the initial development of the CCNC and implementation of the initial project.

### Moi University Clinical and Cognitive Neuroscience Center (MU CCNC) Equipment and Processes Assessed

The MU CCNC was equipped with a 32-channel EEG system from BrainVision (Brain Products, Munich, Germany) and ActiiCAP slim 32-channel electrode set and eight EEG caps (Brain Products GmbH, 2017) for neurophysiological recording, and E-Prime (Psychology Software Tools, Pittsburgh, PA) and MATLAB for stimulus presentation (The MathWorks Inc., 2022); thus, allowing for the recording of ERPs. Paired with this stimulus presentation software, the EEG system can record ERPs during passive or active task conditions or continuously record the electroencephalograph when a participant is at rest. The initial battery of ERP procedures established at the CCNC assessed sensory and cognitive processes: auditory steady state response (ASSR)^16^; dual-click P50 sensory gating^17^; mismatch negativity (MMN)^18^ P300 auditory oddball task^19^; and error-related negativity (ERN).^20^ The CCNC also has capability to assess two neuromotor processes: delay eye blinking conditioning (dEBC),^21^ which utilizes the bioamplifiers to record electromyographic (EMG) recordings on the orbicularis of the eye, and postural sway recorded using an AMTI Accusway (Watertown, MA) force platform.^22^ Electrocardiogram (EKG) is also passively recorded utilizing two bipolar sensors (electrodes) placed on each forearm a few centimeters above the wrist; these recordings may be used to assess heartrate variability and the heartrate evoked potential.^23^ Taken together, these procedures provide an expansive neural assessment of sensory, motor, and cognitive function.

## Considerations for Laboratory Development

The CCNC was established to conduct state-of-the-art research on cognitive processes in children and adults in western Kenya. Located within a public academic medical referral center, the CCNC is uniquely positioned to support research across a wide range of disease and conditions. In addition to research, the center serves as a training platform for undergraduate and master's-level students in psychology and medicine, supporting instruction in medical physiology and cognitive neuroscience. The CCNC will also host investigator-initiated studies led by Moi University faculty and, through partnerships with local faculty investigators, will facilitate national and international research collaborations. Clinical EEGs performed to diagnosed epilepsy take place in a separate clinical laboratory located within the referral hospital campus.

### Location

The CCNC is located at a central location in Moi University's Medical Education Center to maximize accessibility to study participants, faculty, and trainees. The medical campus resides in the fifth largest city in Kenya, Eldoret, and the lab is located within a busy health sciences building along a main road, requiring special attention to minimize acoustic noise from the street, adjacent rooms, and hallways. Standard operating procedures (SOPs) were developed to ensure continuity of training and capacity building across time.

### Travel

Team members from USA traveled to Kenya three times during the five months between delivery and setup of the neurophysiology equipment, training of the laboratory staff, and commencement of data collection. All equipment was carried in durable carry-on and checked luggage on commercial flights by team members to ensure safe and timely arrival. It is useful to have documentation from the partnering universities of the educational and research purposes of the equipment to present to border control agents if necessary. Each visit lasted 10 to 14 days.

Members of the USA research team visit three to four times annually to assist with data quality and processing. Bi-monthly videoconferencing addresses questions and issues that arise pertaining to protocols and equipment.

### Budget

Budget considerations are crucial when creating and maintaining a neuroscience lab, as they can significantly impact the lab's long-term viability and functionality. Major budget items include: neurophysiology/EEG bioamplifiers; at least two computers (eg, a laptop for stimulus presentation and another for neurophysiology recording); stimulus presentation and neurophysiology recording and analysis software; furniture such as tables and chairs; supplies such as EEG caps, re-usable sensors, skin preparation solutions, conductive gel, and data storage media; white noise generator to drowned out ambient acoustic noise; long-term maintenance costs for replenishing supplies, replacing computers, and updating software; and trained personnel to oversee day-to-day operations. The upfront costs for this lab (in 2021) were approximately: USD $70,000 for a 32-channel bio-amplifier system, caps, and software; $4500 for two high-performance laptops, a monitor for visual stimulus presentation, and external hard drives; $1000 for stimulus presentation software and peripherals; and $2500 for office chairs, a barber chair for electrode placement, desks, tables, rolling cart, supplies, and storage cabinets. Additionally, the laboratory space and associated office(s) will either need to be adapted in existing building space or built.

Maintenance may vary according to the level of laboratory utilization. Because the CCNC is housed within the university, core infrastructure expenses (including rent, internet, and electricity) are fully covered. Equipment upgrades are supported through new research grants, as needed. A dedicated, long-term research staff member oversees routine testing, maintenance, and troubleshooting of all equipment to ensure ongoing functionality and timely identification of any issues. These responsibilities require approximately 5% of a full-time individual's effort.

### Electrical Power

A neurophysiology lab requires reliable electrical power, with an earth ground. For the set-up described here, about 12 power outlets are required via three power strips with surge-protection. We selected a rechargeable battery-operated EEG system and laptops so that transient and short-term power loss would not disrupt recordings. Another advantage of battery-operated EEG devices is that they can be disconnected from alternating current (AC) power outlets to reduce the propagation of 50 Hz (Africa) or 60 Hz (North America) electrical line noise during recording. Uninterruptible power supplies (UPSs) can be added to support electrical equipment and reduce the chance of data loss during power interruptions. Finally, when setting up a neurophysiology lab one should position pieces of equipment that utilize AC power as far away as possible from the EEG system so that line noise doesn’t contaminate the EEG recording.

### System Setup

**Figure 1.**
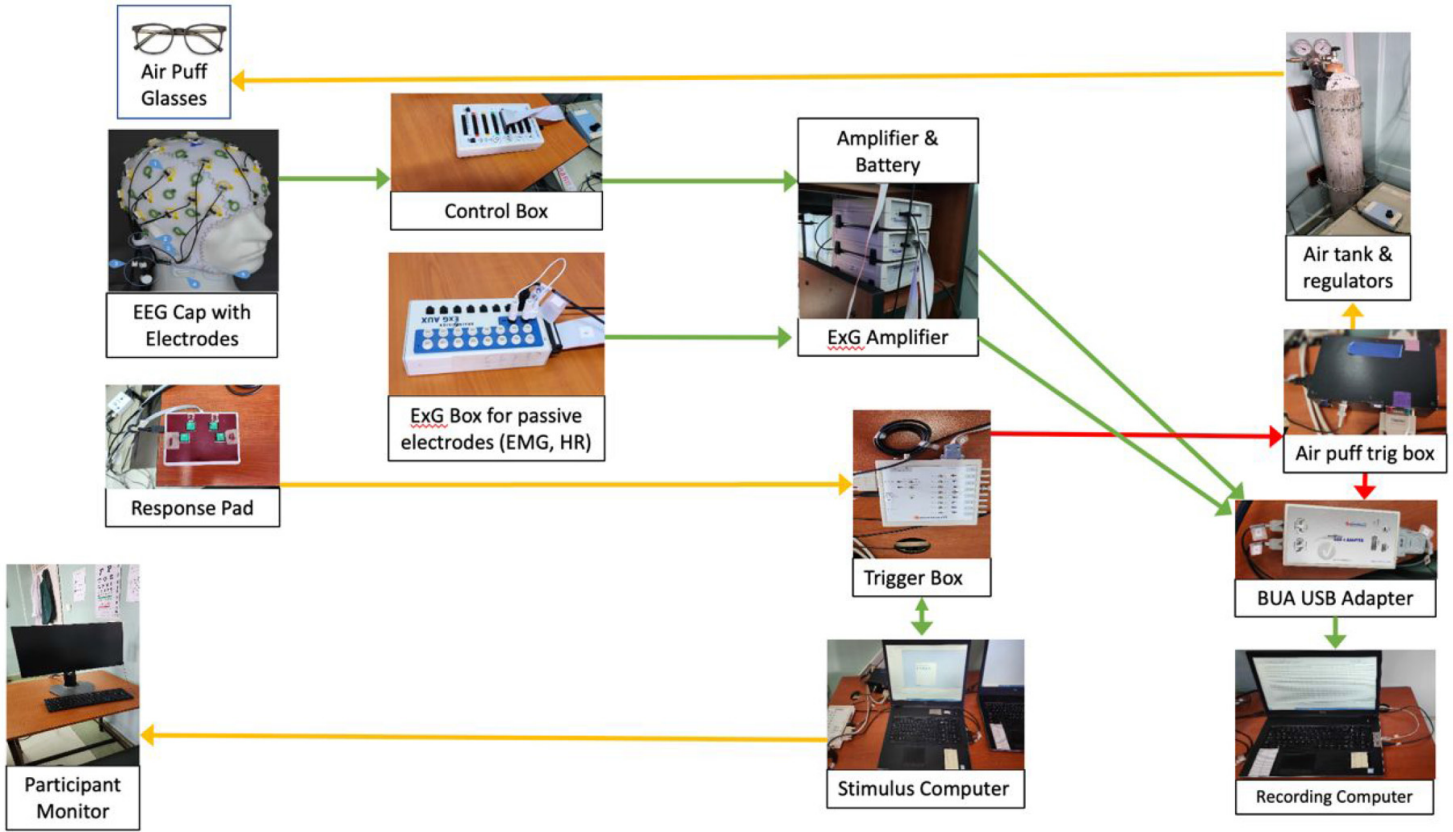
Electrophysiology lab set-up.

The EEG system was placed in a corner of a large room, away from windows and power outlets so that acoustic, electrical, and visual (eg, direct sunlight) noise would be reduced to the extent possible. System components were arranged to utilize cables that were as short as possible and crossed each other as little as possible to minimize the likelihood of electrical noise contamination. The major connections between components are shown below (Figure 1).

**Figure 2.**
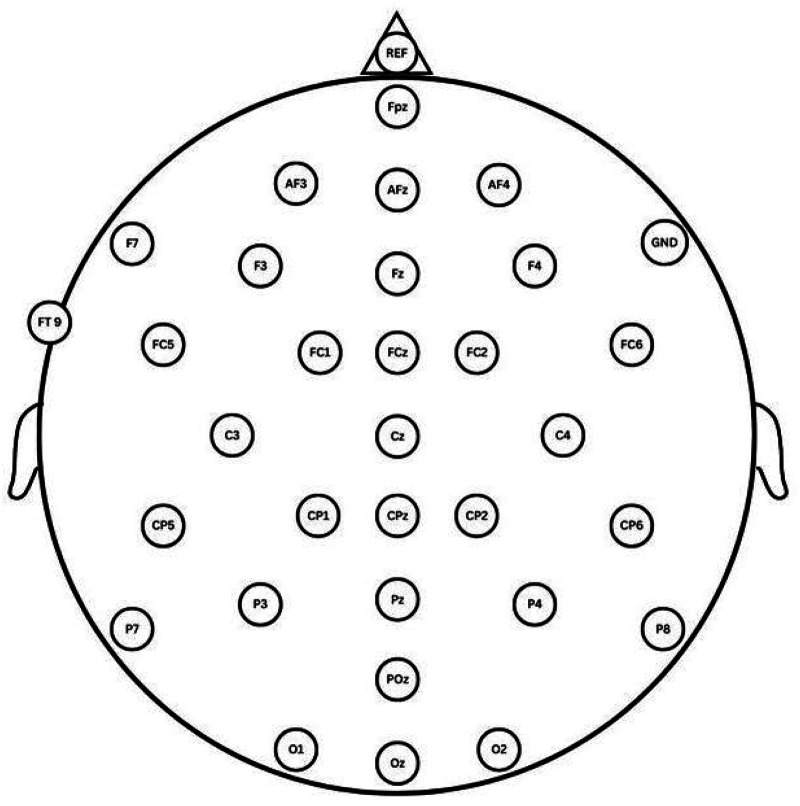
EEG cap configuration utilized.

Special considerations were taken for placement of electrocardiogram (EKG) and EEG sensors on participants, so that they did not need to adjust or remove typical clothing items. For example, sensors to record heart rate (EKG) were placed on each forearm medial to the wrist instead of on the torso and shoulder regions. A 32-lead EEG cap was utilized to position sensors on the scalp following the 10–20 system (Figure 2). This method was selected due to its ability to maximize precision and rigor of the resulting data for hypothesis-driven research, as well as aid to electrode placement with variety of hair styles and textures. Labs should discuss and decide on which sensor locations are best suited to capturing the EEG and ERP signal of interest.^24^ EEG recording was conducted in accordance with established protocols.^25^

### Environmental Sources of Noise

Special consideration was taken to minimize electrical, acoustic, visual, physiological, and motion-based noise. Line noise originates from alternating current (AC) power lines, which in Kenya alternate at 50 cycles per second (50 Hz) at 220 volts (compared, for example, to 110 volts in the USA). To reduce 50 Hz line noise, we selected a battery-powered EEG system that can be disconnected from AC power during recording and positioned away from wall outlets. AC-powered equipment (eg, video monitors) was placed far from EEG devices. A voltage regulator (1000 W) with input/output display ensured compatibility with 110 V devices using a step-down converter. Power needs were calculated using device specifications.

Initial setup revealed high 50 Hz noise, suspected to stem from either a floating electrical ground or ground-loop issues. Removing metal furniture and ensuring sensor leads avoided contact with metal and other cables resolved this. Relocating the participant further from AC sources helped. Additional noise was caused by dry gel increasing sensor impedance. This discovery precipitated new protocols being introduced to meticulously but gently clean sensors to avoid this source of noise.

Impedance refers to the electrical resistance of the scalp sensor, measured in kilo-ohms (kΩ). Maintaining low impedance (<10 kΩ) is critical for EEG data quality. Since perspiration from the head can adversely affect impedances, it is desirable to conduct testing when participants are not perspiring, which was generally not an issue in our temperate setting.

Distractions of any of the five senses can lead to participant movement or cognitive processes related to the distractions and compromise the data quality. To reduce disruptions, windows were kept closed, white noise generators used, and “quiet” signs printed. Visual distractions were controlled by placing the display screen against plain backgrounds in a dim room with sunlight-blocking curtains.

Participant motion can introduce muscle artifacts. Instructions were given to remain still, and staff monitored recordings in real time. A comfortable barber chair supported posture to reduce movement.

Equipment layout was optimized for consistency and data quality. Cables and devices were fixed in place, and battery-powered components were positioned near sensitive amplifiers. EEG sensor leads were routed neatly, avoiding folds or pinches. Amplifiers were placed on rolling carts to facilitate easier setup and cleanup.

### Connectivity

Access to an internet connection is key to maintaining open communication, updating software, and uploading data to the cloud. Since the building is not wired with internet connections, a mobile hotspot was purchased to provide internet connectivity. This feature proved invaluable on several occasions, including when the lab needed to conduct videoconferencing with the EEG system supplier to troubleshoot the environmental electrical noise and with North American colleagues about updating software licenses.

It is important to note that the MU CCNC does not allow the laptop used for stimulus presentation to connect to the internet in order to prevent automatic updates to the acquisition laptop's software. Some software and hardware components might not work if a system update disrupts the way the software interacts with the computer operating system or hardware. Following acquisition, a second laptop may be used for cloud upload with transferred data.

### Data Storage and Backups

Ensuring secure and accurate data storage for complex data files was another important consideration. Google Drive is utilized as a HIPAA compliant cloud service, where only study personnel have access. Utilizing a cloud service allows critical functionality for personnel in different countries to easily access shared materials. A password protected and encrypted hard drive is utilized locally. To prevent data loss, data is immediately backed up and stored in multiple secure locations (eg, Google Drive & hard drives).

### Cultural Considerations

Cultural and language adaptations were necessary. First, each EEG procedure had two language versions created so that instructions could be presented in both in English and Swahili. Second, thicker hair types present challenges in placing the EEG sensors near to the scalp so that the sensor gel forms an electrically conductive bridge between the surface of the scalp and the sensor (see^26,27^ for detailed discussion of this issue). The lab has a wide selection of EEG cap sizes so that a tight-fitting but comfortable cap size can be used to position sensors as close to the scalp as possible. When necessary to position certain areas of the EEG sensor cap closer to the scalp, medical elastic tubular mesh bandage is wrapped around the head and over the surface of the cap to make the cap more secure against the head. Additionally, funds were included in the budget to reimburse participants for a haircut if they preferred to cut their hair prior to their participation in the research, based on information about the placement of the EEG sensors.

### Considerations During a Typical Visit

To ensure precisely timed stimulus presentation, BrainVision's StimTrak (Brain Products, Munich, Germany) was used as a peripheral add-on. The StimTrak detects when auditory and visual stimuli are presented to the participant by intercepting the audio and video signals, then sending a digital signal to the BrainVision recording software. In this manner, the experimenter can track if the triggers generated by the stimulus presentation software and embedded in the EEG consistently align with the actual onset of stimuli being presented to the participant. If not, the experimenter can try to optimize the code to eliminate the lag or use the markers generated by StimTrak embedded in the EEG record to adjust the latency of ERPs during processing of the data.

Given the EEG assessment protocol contained multiple EEG/ERP task procedures, the lab had to consider the cost versus benefit of having participants complete all the procedures in one long testing session versus breaking up the assessments across multiple days. Since some of our participants travel from distant locations, it was decided that one long EEG testing session in a single day would be best for the participants and enhance comparability of data across task procedures. To reduce monotony and fatigue, planned breaks were built into testing sessions and EEG staff were trained to be vigilant to participant energy fluctuations throughout the testing.

### Software Selection

The MU CCNC utilizes E-Prime computer software (Psychology Software Tools, Pittsburgh, PA), MATLAB computer software (The MathWorks Inc., 2022), and BrainVision Recorder (Brain Products, Munich, Germany). In order to make the EEG system as accessible as possible to researchers with little or no coding experience, we selected well-documented and supported graphical user interface (GUI) software. Our group utilizes these programs across two sites in North America and two sites in Africa; thus, it is easy for us to update and test EEG task procedures at any one of our sites and share it widely.

### EEG Setup Training

Staff and a graduate student from the United States systematically trained Kenyan staff in the Eldoret laboratory space in EEG setup, recording, and in how to back up EEG data. Research staff provided an overview of neural sources of human EEG, how it is measured, what can be learned from the signal, as well as what artifacts can contaminate EEG signals, how to recognize them, and how artifacts can be mitigated. The staff learned about equipment function, individual components of the Brain Vision 32-channel EEG system (Brain Products, Munich, Germany), and how to properly connect them. Precautions included not allowing any liquid to touch the system except for conductive gel on sensors, keeping wires free from entanglement and crossing, regularly assessing for loose connections, and keeping the area surrounding the equipment clean and free from food. Approximately 10 sessions were required to achieve consistent, acceptable electrode placement among three EEG laboratory staff using the standard 10–20 system. Fewer sessions would likely be sufficient if a shortcut placement method were used. Practices and procedures for operating BrainVision Recorder, E-Prime, and Matlab software for data collection were also demonstrated. A weeklong training period on EEG best practices was led by a professor with expertise in EEG, a PhD student, and an experienced laboratory manager from the USA. Finally, potential problem scenarios were practiced with the Moi-based research staff to increase awareness of possible threats to stimulus presentation and data collection, increase mastery, and increase self-sufficiency. To maintain consistency and assure self-reliance once USA personnel left, a comprehensive SOPs manual was created that focused on system set up, impedance checks, recording and collecting data, sensor cleaning, aftercare, and sway platform setup. An example of one such SOP is found within Appendix A. Additionally, annual workshops on EEG principles and techniques to aid with sustainability in neurophysiology teaching and research nationwide have been hosted.

### Communication

Regular communication among study personnel has proven to be essential for smooth operation and to work through various issues as they arise. Study collaborators communicate consistently across various mediums, including videoconferencing, email, and hand-held device applications (eg, WhatsApp), with formal meetings bi-monthly. Furthermore, written logs are kept to document data collection issues (eg, impedance problems, observed data artifacts, etc) and to be alert to the need to restock certain supplies.

### Maintenance

To keep equipment and lab space working efficiently, special considerations were taken. Staff were trained to clean and store sensors and maintain a clean workspace. As commercial sensor disinfectants are not readily available in Kenya, the lab was purposefully located near a water source, so that staff can utilize a water and diluted alcohol solution to clean sensors. Disinfecting the reusable EEG sensors and storage in clean containers is important, as reusable sensors are vulnerable to bacteria.^28^ For more information about EEG sensor impedance, see Ferree et al^29^ and Kappenman and Luck.^30^ The lab also utilizes baby soap, which is less abrasive than regular soap, to clean the lycra EEG sensor caps. Due to periods of high humidity in Eldoret, Kenya, plastic containers that seal tightly are used to store the EEG sensors once they are fully cleaned and dried. Prolonged exposure to humidity can decrease the lifespan of the sensors.

### Open Science Resources

While the lab chose to use widely available proprietary software, open source software such as Python programming language could have been utilized for creation of scripts responsible for stimulus presentation.^31^ As open-source programs are readily available, use of such software could be of benefit to labs which have personnel with coding experience. The use of open-source programs can reduce financial burden and open access to a community of users for assistance; however, it can require significant knowledge of coding. Proprietary software, such BrainVision's acquisition and analysis software and E-Prime, requires a minimal level of coding ability, but can be costly. Labs must weigh such pros and cons. Training staff to code beforehand or hiring personnel with coding abilities would be of benefit for labs aiming to utilize open-source software. Further, there are growing open-source repositories of paradigms for EEG research such as the ERP CORE, which contains protocols for several common ERPs.^32^ As open-access resources are increasingly available on the internet, the creation of EEG labs will be possible with less financial investment and greater support from other users.

## Conclusions

Through the process of building human neurophysiology research infrastructure in a resource-constrained environment, we gained invaluable insights and learned critical lessons described here, which we hope will be of benefit to others who undertake similar projects. Beyond securing funding, one of the most significant challenges in establishing the laboratory was minimizing acoustic, visual, and electrical noise in this environment- an essential step in ensuring the collection of high-quality data with highly sensitive equipment. Through a bi-national and bi-university collaboration, we addressed potential barriers and established a world-class human neurophysiology lab in an environment where extraordinary research can be conducted. The development of infrastructure to support and advance human clinical and cognitive neuroscience research has empowered our team of faculty, students, and staff to lead new initiatives, such as managing a comprehensive pilot study, developing a graduate program curriculum, and organizing multiple research meetings and workshops featuring prominent clinical and basic researchers from across Kenya.

It is crucial that labs consider the potential pitfalls that could arise during all phases of lab setup because proper planning and early precautionary steps can ensure high quality data from the outset and greatly limit the number of issues that arise later. Apart from this work, various published works have written on best practices for setting up an EEG lab and potential issues that should be taken into consideration.^24,33^ These guides should serve as a foundation for lab development, with the considerations we have described as a supplement when developing a lab in LMICs. While many considerations were apparent before our lab was set up, additional factors became apparent during the lab setup and testing phase.

To our knowledge, this human neurophysiology research laboratory is the first of its kind at a public academic institution in sub-Saharan Africa. This newly established clinical and cognitive neuroscience research infrastructure holds immense promise for promoting equity in research utilizing neurophysiological, psychological, and cognitive methods. This infrastructure facilitates the generation and exploration of research questions by local scientists, clinicians, students, and community members who are best situated to identify and prioritize physical and mental health needs. The establishment of this infrastructure, coupled with capacity-building efforts to provide intensive training in these research techniques, is poised to enhance neurophysiology, cognition, and neurodevelopmental outcomes throughout western Kenya and across the entire continent.

## Supplemental Material

sj-doc-1-eeg-10.1177_15500594261432722 - Supplemental material for Development of a Human Electrophysiology Laboratory to Advance Cognitive Neuroscience in Western Kenya Supplemental material, sj-doc-1-eeg-10.1177_15500594261432722 for Development of a Human Electrophysiology Laboratory to Advance Cognitive Neuroscience in Western Kenya by Amira Nafiseh, Jaime Morales, Mark Nyalumbe, Eren Oyungu, David Ayuku, Amanda R Bolbecker, William P Hetrick and Megan S McHenry in Clinical EEG and Neuroscience

## References

[1] Nations U . Population n.d. https://www.un.org/en/global-issues/population.

[2] Singh I . Neuroscience for global mental health. Cerebrum. 2020;2020:8-20. PMC7664820 33216831

[3] Hilal S Brayne C . Epidemiologic trends, social determinants, and brain health: The role of life course inequalities. Stroke. 2022;53(2):437-443. 35000426 10.1161/STROKEAHA.121.032609

[4] Moore TG McDonald M Carlon L O'Rourke K . Early childhood development and the social determinants of health inequities. Health Promot Int. 2015;30(suppl_2):ii102-iii15. 10.1093/heapro/dav031 26420806

[5] Rappaport SM Smith MT . Epidemiology. Environment and disease risks. Science. 2010;330(6003):460-461. 20966241 10.1126/science.1192603 PMC4841276

[6] Maina MB Ahmad U Ibrahim HA , et al. Two decades of neuroscience publication trends in Africa. Nat Commun. 2021;12(1):3429. 34103514 10.1038/s41467-021-23784-8 PMC8187719

[7] McLane HC Berkowitz AL Patenaude BN , et al. Availability, accessibility, and affordability of neurodiagnostic tests in 37 countries. Neurology. 2015;85(18):1614-1622. 26446063 10.1212/WNL.0000000000002090 PMC4642148

[8] Vegda H Krishnan V Variane G Bagayi V Ivain P Pressler RM . Neonatal seizures—perspective in low-and middle-income countries. Indian J Pediatr. 2022;89(3):245-253. 35050459 10.1007/s12098-021-04039-2 PMC8857130

[9] Bhavnani S Parameshwaran D Sharma KK , et al. The acceptability, feasibility, and utility of portable electroencephalography to study resting-state neurophysiology in rural communities. Front Hum Neurosci. 2022;16:802764. 35386581 10.3389/fnhum.2022.802764 PMC8978891

[10] Light GA Williams LE Minow F , et al. Electroencephalography (EEG) and event-related potentials (ERPs) with human participants. Curr Protoc Neurosci. 2010;52:6-25. 10.1002/0471142301.ns0625s52 PMC2909037 20578033

[11] Simpkin V Namubiru-Mwaura E Clarke L Mossialos E . Investing in health R&D: Where we are, what limits us, and how to make progress in Africa. BMJ Glob Health. 2019;4(2):e001047. 10.1136/bmjgh-2018-001047 PMC6407556 30899571

[12] Center NFI. Global Brain Disorders Research 2023. https://www.fic.nih.gov/Programs/Pages/brain-disorders.aspx.

[13] Ledwidge P Foust J Ramsey A . Recommendations for developing an EEG laboratory at a primarily undergraduate institution. J Undergrad Neurosci Educ. 2018;17(1):10-19. PMC6312138 30618494

[14] Turissini M Mercer T Baenziger J , et al. Developing ethical and sustainable global health educational exchanges for clinical trainees: Implementation and lessons learned from the 30-year academic model providing access to healthcare (AMPATH) partnership. Ann Glob Health. 2020;86(1):137. 33178558 10.5334/aogh.2782 PMC7597575

[15] Kwobah E Jaguga F Robert K Ndolo E Kariuki J . Efforts and challenges to ensure continuity of mental healthcare service delivery in a low resource settings during COVID-19 pandemic-A case of a Kenyan referral hospital. Front Psychiatry. 2020;11:588216. 33519544 10.3389/fpsyt.2020.588216 PMC7840829

[16] Rass O Forsyth JK Krishnan GP , et al. Auditory steady state response in the schizophrenia, first-degree relatives, and schizotypal personality disorder. Schizophr Res. 2012;136(1-3):143-149. 22285558 10.1016/j.schres.2012.01.003 PMC3298621

[17] Brenner CA Kieffaber PD Clementz BA , et al. Event-related potential abnormalities in schizophrenia: A failure to “gate in” salient information? Schizophr Res. 2009;113(2-3):332-338. 19628376 10.1016/j.schres.2009.06.012 PMC2820396

[18] Lundin NB Burroughs LP Kieffaber PD Morales JJ O'Donnell BF Hetrick WP . Temporal and spectral properties of the auditory mismatch negativity and P3a responses in schizophrenia. Clin EEG Neurosci. 2023;54(4):409-419. 35341344 10.1177/15500594221089367

[19] O'Donnell BF Vohs JL Hetrick WP Carroll CA Shekhar A . Auditory event-related potential abnormalities in bipolar disorder and schizophrenia. Int J Psychophysiol. 2004;53(1):45-55. 15172135 10.1016/j.ijpsycho.2004.02.001

[20] Zirnheld CAC PJ Kieffaber PD O'Donnell BF Shekhar A Hetrick WP . Haloperidol impairs learning and error-related negativity in humans. J Cogn Neurosci. 2004;16(6):1098-1112. 15298795 10.1162/0898929041502779

[21] Forsyth JK Bolbecker AR Mehta CS , et al. Cerebellar-dependent eyeblink conditioning deficits in schizophrenia spectrum disorders. Schizophr Bull. 2010;38(4):751-759. 21148238 10.1093/schbul/sbq148 PMC3406528

[22] Bolbecker AR Apthorp D Bartolomeo LA O'Donnell BF Hetrick WP . Postural sway in first-degree relatives of individuals with schizophrenia. Schizophr Res. 2021;228:319-321. 33497906 10.1016/j.schres.2020.12.024

[23] Brian F O'Donnell KMW Hetrick WP . Psychophysiology of mental health. Encyc Mental Health. 2023:894-904.

[24] Hari R Puce A Hari M , et al. 3Introduction. In: Hari R Puce A , eds. MEG-EEG Primer. Oxford University Press; 2017.

[25] Farrens JL Simmons AM Luck SJ Kappenman ES . Electroencephalogram (EEG) recording protocol for cognitive and affective human neuroscience research. Protocol Exchange. 2020.

[26] Etienne A Laroia T Weigle H , et al. Novel electrodes for reliable EEG recordings on coarse and curly hair. Annu Int Conf IEEE Eng Med Biol Soc. 2020;2020:6151-6154. 33019375 10.1109/EMBC44109.2020.9176067

[27] Choy T Baker E Stavropoulos K . Systemic racism in EEG research: Considerations and potential solutions. Affect Sci. 2022;3(1):14-20. 36042782 10.1007/s42761-021-00050-0 PMC9383002

[28] Albert NM Bena JF Ciudad C , et al. Contamination of reusable electroencephalography electrodes: A multicenter study. Am J Infect Control. 2018;46(12):1360-1364. 29997036 10.1016/j.ajic.2018.05.021

[29] Ferree TC Luu P Russell GS Tucker DM . Scalp electrode impedance, infection risk, and EEG data quality. Clin Neurophysiol. 2001;112(3):536-544. 11222977 10.1016/s1388-2457(00)00533-2

[30] Kappenman ES Luck SJ . The effects of electrode impedance on data quality and statistical significance in ERP recordings. Psychophysiology. 2010;47(5):888-904. 20374541 10.1111/j.1469-8986.2010.01009.x PMC2902592

[31] Rossum GV Drake FL . Python 3 Reference Manual: CreateSpace; 2009.

[32] Kappenman ES Farrens JL Zhang W Stewart AX Luck SJ . ERP CORE: An open resource for human event-related potential research. Neuroimage. 2021;225:117465. 33099010 10.1016/j.neuroimage.2020.117465 PMC7909723

[33] Kappenman ES Luck SJ . Best practices for event-related potential research in clinical populations. Biol Psychiatry Cogn Neurosci Neuroimaging. 2016;1(2):110-115. 27004261 10.1016/j.bpsc.2015.11.007 PMC4797328

