# Supplementary material for: Development of a Human Electrophysiology Laboratory to Advance Cognitive Neuroscience in Western Kenya
Source: Clin EEG Neurosci. 2026 May 11;57(4):321–8. doi: 10.1177/15500594261432722 (PMC13242544; doi:10.1177/15500594261432722)
Supplement: sj-doc-1-eeg-10.1177_15500594261432722 - Supplemental material for Development of a Human Electrophysiology Laboratory to Advance Cognitive Neuroscience in Western Kenya [file sj-doc-1-eeg-10.1177_15500594261432722.doc]

**Protocol for Study Research Assistant/Intern**

1. *General procedures*:
   1. The day before a clinic day, allocate individual participants’ reimbursement (KES divided per participant expected)
   2. Ensure everything required for the day is ready and set including space if needed - booking with research office, for seeing the participants in a private and confidential way.
   3. Confirm availability of the correct versions of the following documents. Confirm that you have multiple copies of each form.
2. Consent/Assent forms in English and Swahili
3. Data Collection Tools: All study questionnaires for the visit.
   1. Keep study folder complete, clean and organized
   2. Complete data collection per the study protocol.
   3. Document responses on hard copies of the data collection tools.
   4. All research files are to be kept locked in a cabinet in the research office. Each physical form filled will be scanned and saved in an electronic encrypted file.
   5. Data should be entered into the TABIRI/Neuro EEG Study REDCap database by end of day if possible or by end week. Additional study personnel are to cross-check data entered on a weekly basis (Please see data cross checking SOP). Daily updates should include data entry and cross checking updates if done for the day.
   6. Planning on reviewing entered data and compile weekly report of those entered and cross-checked into REDCap. *Refer to Data management SOP*
   7. Project Coordinator to send weekly update to PIs:
4. *Specific Procedures:*
   1. The day before the scheduled study visit, review the anticipated number of participants. The RA will call the anticipated participant to remind them of their upcoming visit.
   2. On the clinic day, attend clinic at all times to screen un-scheduled patients for eligibility. Additionally, discuss with registration personnel at the clinic/mentor mother and ask to be notified of patients who arrive without an appointment
   3. Initiate Consent/Assent processes for the patient/participant. Collect data from the participants using data collection tools and record the next clinic visit date.
   4. Finalize payment to the participant and record in appropriate patient reimbursement form.

***SOP Reviewed and Approved:***

*Date SOP Approved:*

*Date of Next Review:*

***I hereby attest that I have read and understood the above SOP and agree to abide by its contents:***

| **Role** | **Name** | **Signature** | **Date** |
| --- | --- | --- | --- |
| Principal Investigator |  |  |  |
| Co-Principal Investigator |  |  |  |
| Project Coordinator |  |  |  |
| Study Research Assistant |  |  |  |
| Research Intern |  |  |  |
| Research Intern |  |  |  |

**-2-**
